# CCN3 promotes epithelial-mesenchymal transition in prostate cancer via FAK/Akt/HIF-1α-induced twist expression

**DOI:** 10.18632/oncotarget.20171

**Published:** 2017-08-10

**Authors:** Po-Chun Chen, Huai-Ching Tai, Tien-Huang Lin, Shih-Wei Wang, Chih-Yang Lin, Chia-Chia Chao, Hong-Jeng Yu, Yu-Chieh Tsai, Yu-Wei Lai, Chiao-Wen Lin, Chih-Hsin Tang

**Affiliations:** ^1^ Graduate Institute of Biomedical Science, China Medical University, Taichung, Taiwan; ^2^ Department of Medical Research, Chung Shan Medical University Hospital, Chung Shan Medical University, Taichung, Taiwan; ^3^ Department of Biotechnology, College of Health Science, Asia University, Taichung, Taiwan; ^4^ Department of Urology, National Taiwan University Hospital, Taipei, Taiwan; ^5^ Department of Urology, Fu-Jen Catholic University Hospital, New Taipei City, Taiwan; ^6^ School of Medicine, Fu-Jen Catholic University, New Taipei City, Taiwan; ^7^ Department of Urology, Buddhist Tzu Chi General Hospital Taichung Branch, Taichung, Taiwan; ^8^ School of Post-Baccalaureate Chinese Medicine, Tzu Chi University, Hualien, Taiwan; ^9^ Department of Medicine, Mackay Medical College, New Taipei City, Taiwan; ^10^ Department of Respiratory Therapy, College of Medicine, Fu Jen Catholic University, New Taipei City, Taiwan; ^11^ Department of Oncology, National Taiwan University Hospital, Taipei, Taiwan; ^12^ Division of Urology, Taipei City Hospital Renai Branch, Taipei, Taiwan; ^13^ Department of Urology, National Yang-Ming University School of Medicine, Taipei, Taiwan; ^14^ Institute of Oral Sciences, Chung Shan Medical University, Taichung, Taiwan; ^15^ Department of Dentistry, Chung Shan Medical University Hospital, Taichung, Taiwan; ^16^ Department of Pharmacology, School of Medicine, China Medical University, Taichung, Taiwan

**Keywords:** CCN3, epithelial-mesenchymal transition, prostate cancer, HIF-1α

## Abstract

Epithelial-mesenchymal transition (EMT) has received considerable attention as a conceptual paradigm for explaining metastatic behavior during cancer progression. NOV/CCN3 is a matrix-associated protein involved in many cellular functions. Previous studies have shown that CCN3 expression is upregulated in prostate cancer (PCa) cells and in PCa patients. In this study, we have provided evidence of tumor promoting effects of CCN3, which includes induction of epithelial-to-mesenchymal transition (EMT) and tumor metastasis. We used an orthotopic *in vivo* model to demonstrate the prometastatic effects of CCN3. Overexpression or knockdown of CCN3 changed the EMT phenotype in PCa cells. Moreover, treatment with recombinant CCN3 promoted EMT in PCa cells. We also found that CCN3 may promote EMT by activating the FAK/Akt/HIF-1α pathway and this activation is responsible for Twist expression. IHC staining confirmed a positive correlation between the expression of CCN3, Twist, and tumor stage in PCa tissue. Our findings provide insight into the involvement of CCN3 in the EMT regulation of prostate cancer. CCN3 is a promising molecular target that may contribute to a novel therapeutic strategy against metastatic PCa.

## INTRODUCTION

Prostate cancer (PCa) is the most commonly diagnosed malignancy in the United States and other Western countries [1]. In the early stages of prostate cancer, surgery is the most frequent therapeutic intervention. In advanced disease, more systemic interventions are required to inhibit the growth and spread of secondary metastases.

Cancer metastasis is a critical step in tumor progression and the major cause of mortality for patients with cancer. This process comprises several steps by which cells detach from the primary tumor and form a secondary tumor at a distant site [2]. Epithelial-to-mesenchymal transition (EMT) has received considerable attention as a conceptual paradigm that may explain invasive and metastatic behavior during cancer progression [3]. During this process, epithelial cells lose their polarity and are converted to a mesenchymal phenotype [4]. A hallmark of EMT is the loss of epithelial characteristics, such as decreasing expression of E-cadherin and other cell adhesion molecules, and increasing expression of the mesenchymal marker vimentin. EMT-activating transcription factors such as Twist, Snail, Slug, ZEB1 and ZEB2 orchestrate the EMT process and promote the early steps of metastasis, which consist mainly of local invasion and subsequent dissemination of tumor cells to distant sites [5]. These transcription factors repress E-cadherin expression through binding to the E-box in the E-cadherin gene promoter, which in turn promotes EMT [6–10]. Substantial evidence shows that PCa progression exhibits EMT-like states, characterized by changes in the expression of various markers such as E-cadherin and vimentin, which are associated with invasive behavior [11].

The Nephroblastoma overexpressed (*NOV/CCN3*) gene was first discovered in chicken myeloblastosis-associated virus-induced nephroblastomas [12]. The *NOV* gene encodes a secreted protein that interacts with the extracellular matrix (ECM) and thereby regulates many cellular functions, including cell division, chemotaxis, apoptosis, adhesion, motility, and ion transport [13]. Previous studies have shown that CCN3 expression is upregulated in PCa cells and PCa patients [14], which suggests that CCN3 has a role in prostate tumorigenesis [15]. CCN3 is a multifunctional cytokine that signals between the cell and the ECM. Recent studies have shown a correlation between CCN3 expression and tumor progression in many cancers [16–18], and research suggests that CCN3 may increase the migration of PCa cells by influencing ICAM-1 expression [19]. It is known that CCN3 promotes PCa bone metastasis by modulating the tumor–bone microenvironment [20]. In the present study, we show that CCN3 promotes EMT in tumors and that this activity is regulated by the FAK/Akt/HIF-1α signaling pathway. Analysis of clinical PCa specimens also reveals a positive correlation between CCN3 and Twist expression. This study provides a novel insight into the role of CCN3 in the initiation of metastasis through the modulation of EMT.

## RESULTS

### Knockdown of CCN3 expression inhibits PCa metastasis in the orthotopic model

**Figure 1:**
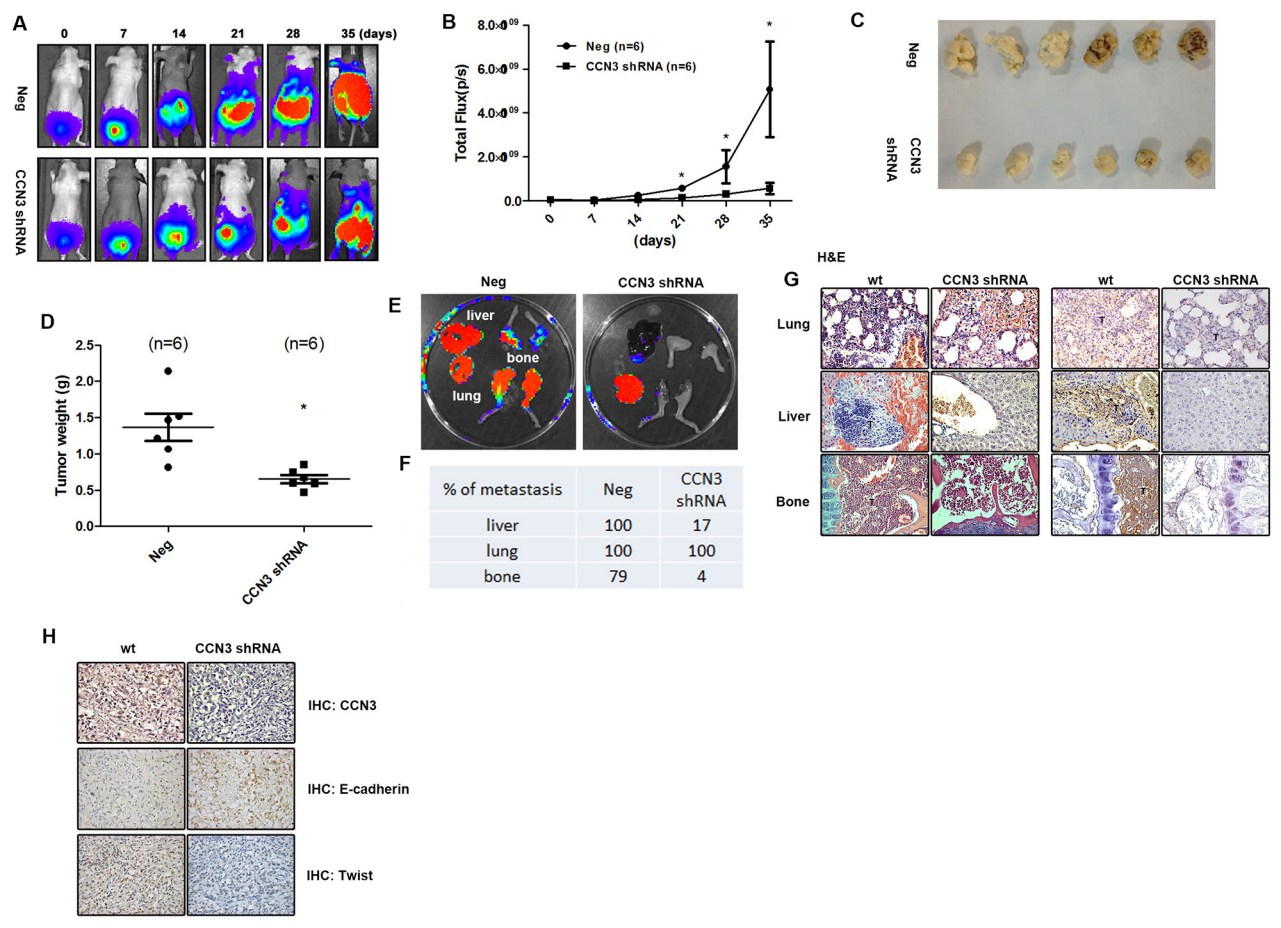
CCN3 is required for metastasis of PCa cells in mouse orthotopic model **(A and B)** CCN3 shRNA (CCN3 shRNA), and control vector PC3 cells (Neg) which stably expressed luciferase were used. The prostates of male nu/nu mice (6–8 weeks old) were exposed by midventral incision and injected with 5 × 10^5^ cells suspended in 50 μL culture media. One week after injection, surgical staples were removed, and the tumor growth and local metastasis were monitored using IVIS Imaging System. The panels depict quantification of fluorescence imaging data acquired at day 1, 7, 14, 21, 28, and 35. **(C and D)** The orthotopic model of nu/nu mice was used. The mice were sacrificed 28 days later, and the tumors were collected from injection site. The tumors were weighed and photographed. **(E)** The orthotopic model of nu/nu mice was set up. The mice were sacrificed 28 days later and their lungs, livers, and legs were dissected and monitored using IVIS Imaging System. **(F)** Quantification of fluorescence imaging data acquired by IVIS Imaging System in (E). **(G)** Left, hematoxylin and eosin (H&E) staining of lung, liver and limb from a control and CCN3 shRNA mice. Tumors were indicated as T. Right, the lung, liver, and limb specimens from sacrificed mice were stained with CCN3 antibody. **(H)** The tumor specimens from sacrificed mice were stained with CCN3, E-cadherin and N-cadherin antibodies. The stained specimens were photographed by optical microscope. Results are expressed as the mean ± S.E.M. *p < 0.05 compared with day 0.

Our previous study describes the role played by CCN3 in enhancing the migration of PCa cells and disease progression[19]. We therefore sought to elucidate the role of CCN3 in PCa metastasis in an orthotopic PCa model. We found that PC3 cells stably expressing CCN3 shRNA showed decreased tumor growth and metastasis (Figure 1A–1D). Interestingly, the metastasis of CCN3 shRNA PC3 cells was dramatically abolished, especially bone metastasis, which has been proposed to be the major cause of mortality in PCa (Figure 1E–1G). A vast amount of evidence has shown that PCa cells exhibit EMT-like states, characterized by changes in the expression of various markers, such as E-cadherin and vimentin, which are associated with invasive behavior [11]. We therefore analyzed the expression levels of EMT markers in tumor specimens. We found that E-cadherin and Twist expression correlated with CCN3 expression in tumor specimens (Figure 1H). These results show that CCN3 serves as a critical regulator of PCa metastasis *in vivo* and correlates with the EMT status.

### CCN3 expression is associated with the mesenchymal phenotype in PCa cell lines

**Figure 2:**
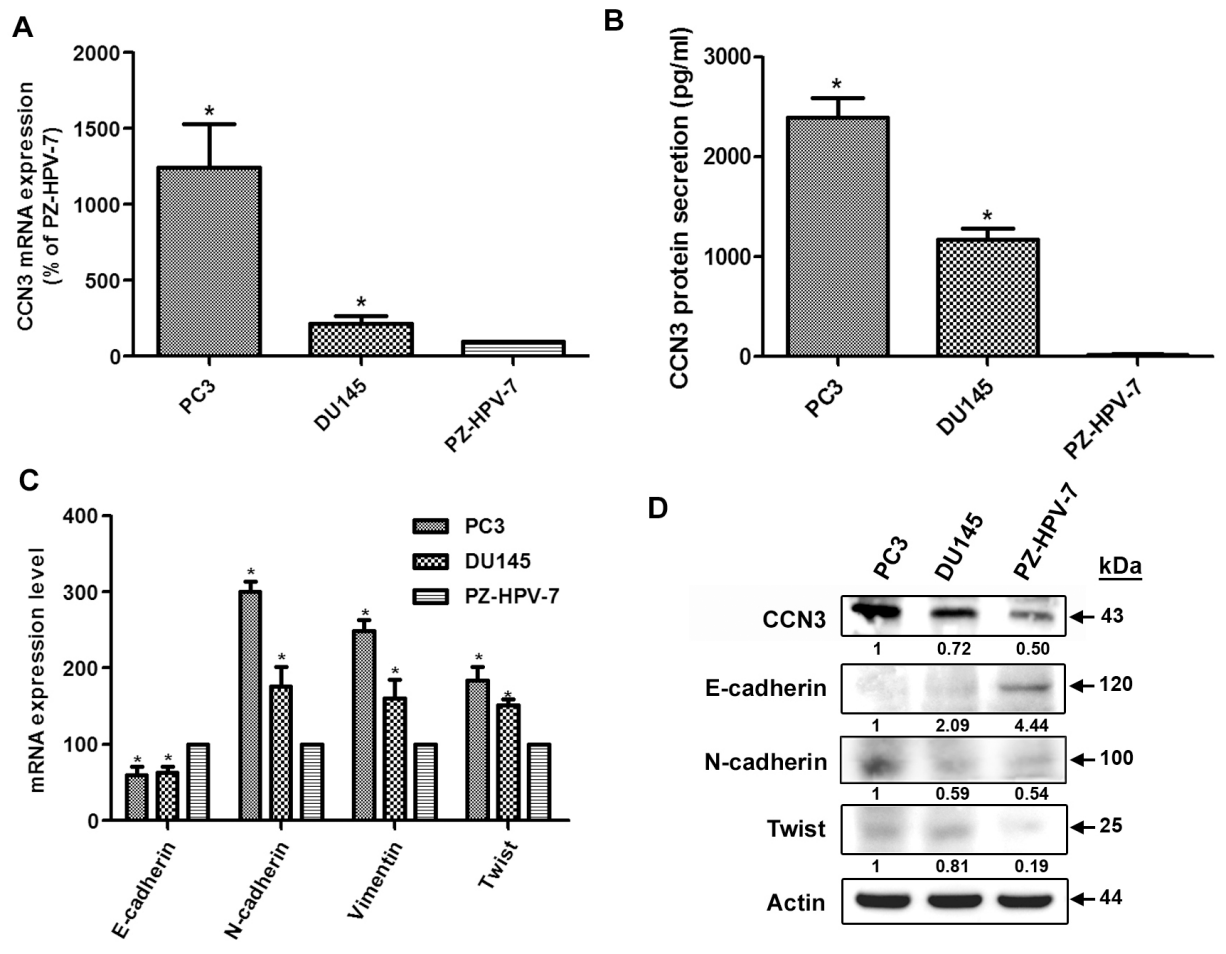
CCN3 is associated with mesenchymal phenotype in PCa cell lines **(A)** Total RNA was extracted from PC3, DU145, and PZ-HPV-7 cells, and CCN3 expression levels were examined by qPCR. **(B)** CCN3 protein secretion in medium from PC3, DU145, and PZ-HPV-7 cells was examined by ELISA. **(C)** Total RNA was extracted from PC3, DU145, and PZ-HPV-7 cells, and the expression levels of E-cadherin, N-cadherin, vimentin, and Twist were examined by qPCR. **(D)** Total protein was extracted from PC3, DU145, and PZ-HPV-7 cells, and the expression levels of CCN3, E-cadherin, N-cadherin, and Twist were examined by western blot analysis. Results are expressed as the mean ± S.E.M. *p < 0.05 compared with PZ-HPV-7 group.

To investigate the role of CCN3 in the EMT process, we examined the correlation between CCN3 expression and EMT markers in PCa cell lines. Interestingly, EMT marker expression patterns were very closely correlated to CCN3 expression. The most aggressive cell line, PC3, expressed the highest levels of CCN3 mRNA and protein (Figure 2A, 2B and 2D). CCN3 expression correlated positively with mesenchymal markers (N-cadherin, vimentin and Twist) but not with the epithelial marker E-cadherin (Figure 2C and 2D). These results suggest that CCN3 expression is positively correlated with the EMT process.

### Overexpression or knockdown of CCN3 affects EMT status in PCa cells

**Figure 3:**
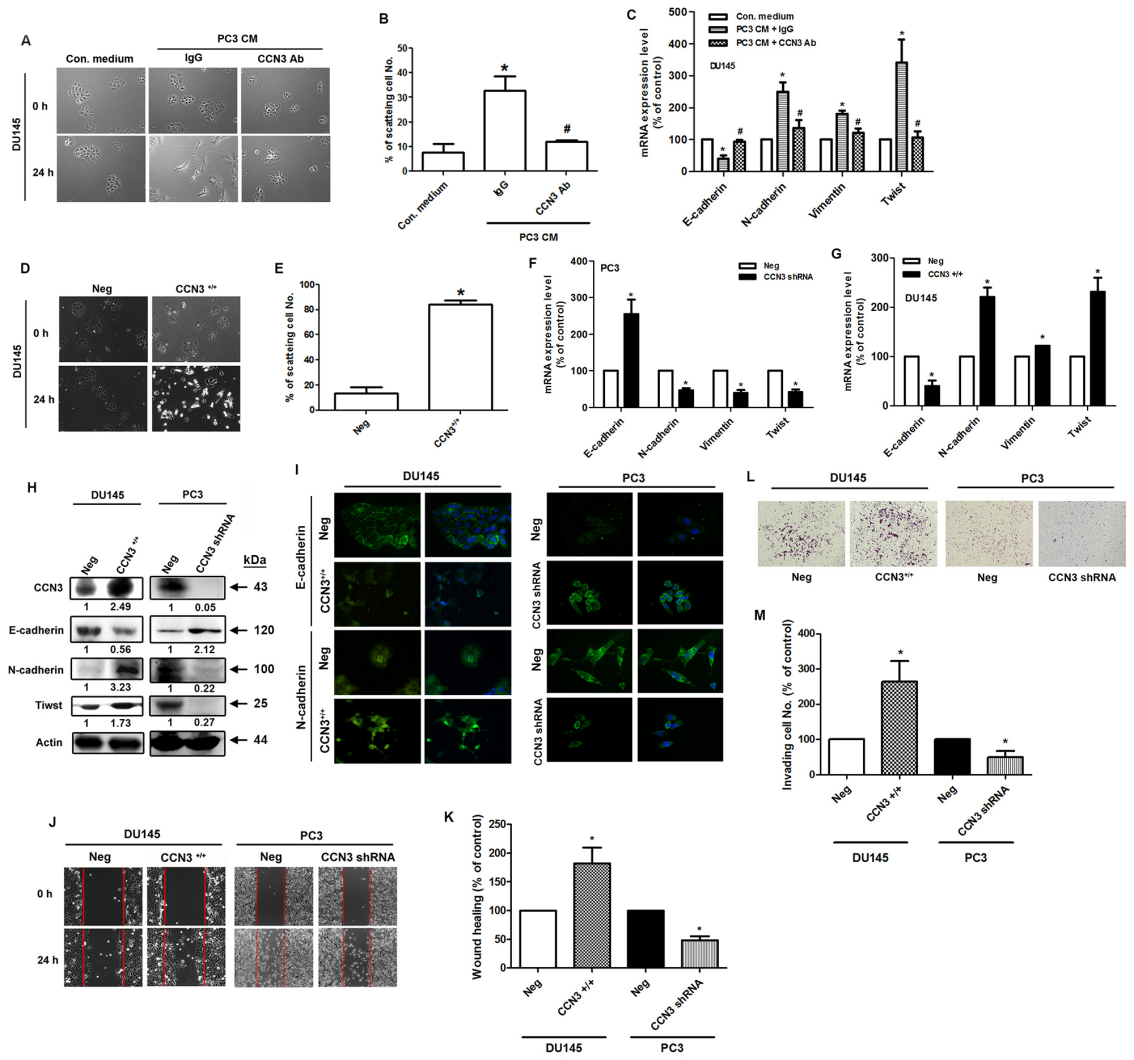
Overexpression or knockdown of CCN3 affects EMT in PCa cells **(A)** The scatter assay was performed on DU145 cells. The cells were incubated with PC3-CM, combined with IgG control or CCN3 neutralizing antibody (1 μg/mL). After 24 h, the DU145 cells were photographed to observe their scattering phenotype. **(B)** The quantitative analysis of scatter assay which was performed in Figure 3A. **(C)** The DU145 cells were treated as described (Figure 2A), total mRNA was extracted and the expression levels of E-cadherin, N-cadherin, vimentin, and Twist were determined by qPCR. **(D)** DU145 cells stably expressing the CCN3 overexpression vector (CCN3^+^/^+^) or control vector (Neg) were established. The scatter assay was performed on DU145 stable cells and the cells were photographed to examine their scattering and morphology. **(E)** The quantitative analysis of scatter assay which was performed in Figure 3D. **(F and G)** DU145 and PC3 cells stably expressing the CCN3 overexpression (CCN3^+^/^+^), CCN3 shRNA vectors (CCN3 shRNA), or control vector (Neg) were established. Total RNA was extracted, and the expression levels of E-cadherin, N-cadherin, vimentin, and Twist were assessed by qPCR. **(H)** Total proteins were extracted from CCN3^+^/^+^ DU145 cells, CCN3 shRNA PC3 cells, and control vector cells. The expression levels of CCN3, E-cadherin, N-cadherin, and Twist were assessed by western blot. Actin was used as internal control. **(I)** CCN3^+^/^+^ DU145 cells, CCN3 shRNA PC3 cells and control vector cells were seeded on glass coverslips. The cells were stained with E-cadherin and N-cadherin antibodies and analyzed by fluorescence microscopy. Nuclei were counterstained with DAPI. Representative microscopy images are shown. **(J and K)** Wound healing assay was performed on DU145 and PC3 stable cells. The migrating cells were photographed and counted. **(L and M)** Cell invasion assay was performed on DU145 and PC3 stable cells. The invading cells were photographed and counted. Results are expressed as the mean ± S.E.M. *p < 0.05 compared with Neg group.

We next tested the EMT-promoting effects of CCN3 by incubating DU145 PCa cells with PC3-conditioned media (CM). The HGF-induced DU145 cell scatter assay has been used previously to mimic the EMT phenomenon [21]. We found that incubation with PC3 CM induced the scattering of DU145 cells and this effect was dramatically abolished by pretreatment with CCN3 neutralizing antibody (Figure 3A and 3B). Moreover, DU145 cells incubated with PC3 CM showed decreased epithelial marker expression (E-cadherin), but increased expression of mesenchymal markers (N-cadherin, vimentin and Twist). These changes in EMT marker expression were reversed by pretreatment with the CCN3 neutralizing antibody (Figure 3C). To further examine the role of CCN3 in EMT, we stably transfected the less malignant DU145 cells with the CCN3 overexpression vector and the highly malignant PC3 cells with the CCN3 knockdown vector. As expected, overexpression of CCN3 improved cell scattering in DU145 cells (Figure 3D and 3E). EMT marker expression was also altered by CCN3 overexpression or knockdown. Overexpression of CCN3 induced the EMT-promoting phenotype in DU145 cells. In contrast, knockdown of CCN3 in PC3 cells showed reversion of EMT phenotype (Figure 3F–3I). EMT has been linked to increased migration and invasiveness in the context of cancer [22]. Results of a wound healing assay demonstrated increased migratory potential of CCN3-overexpressing DU145 cells and decreased migration of CCN3 knockdown PC3 cells (Figure 3J and 3K). Similar results were obtained from an *in vitro* cell invasion assay performed in these stable clones (Figure 3L and 3M). In summary, these results suggest that CCN3 plays a critical role in inducing EMT processes in PCa cells.

### CCN3 directly promotes the EMT process in PCa cells

**Figure 4:**
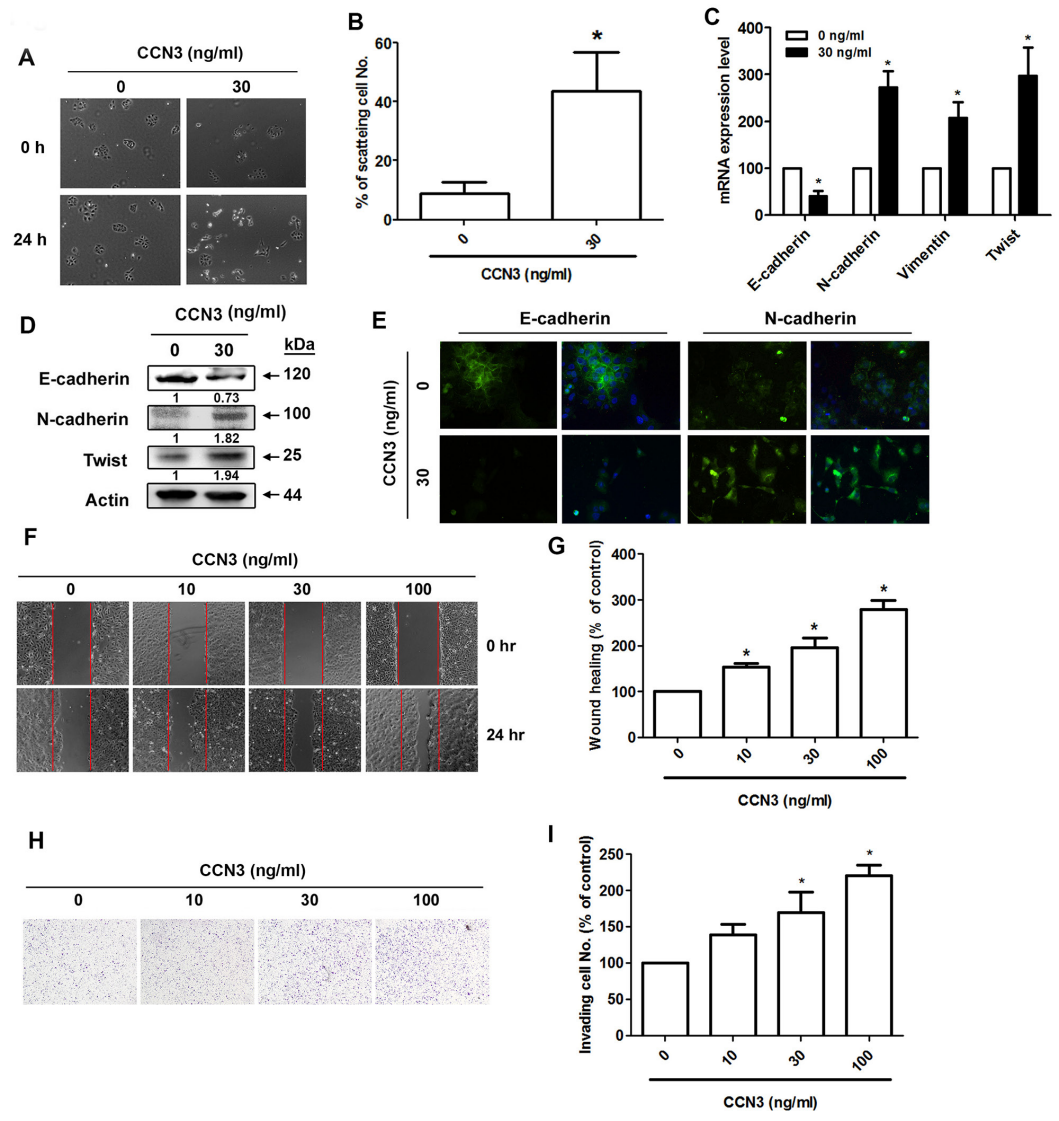
CCN3 directly induces EMT in PCa cells **(A)** DU145 cells were incubated with CCN3 (30 ng/mL) for 24 h and then photographed to examine the scattering and morphology. **(B)** The quantitative analysis of scatter assay which was performed in Figure 3A. **(C** and **D)** DU145 cells were incubated with CCN3 (30 ng/mL) for 24 h and total RNA and protein were extracted. The expression levels of E-cadherin, N-cadherin, vimentin, and Twist were evaluated by qPCR and western blot analysis. **(E)** DU145 cells were incubated with CCN3 (30 ng/mL) for 24 h, the cells were stained with E-cadherin and N-cadherin antibodies and analyzed by fluorescence microscopy. Nuclei were counterstained with DAPI. Representative microscopy images are shown. **(F** and **G)** DU145 cells were incubated with CCN3 (30 ng/mL) for 24 h and *in vitro* migration was measured using wound healing assay. The migrating cells were photographed and counted. **(H** and **I)** DU145 cells were incubated with CCN3 (30 ng/mL) for 24 h and cell invasion assay was performed. The invading cells were photographed and counted. Results are expressed as the mean ± S.E.M. *p < 0.05 compared with control.

To further elucidate the role of CCN3 in EMT induction, we treated PCa cells with recombinant CCN3 (30 ng/mL), which induced DU145 cell scattering (Figure 4A and 4B). Induction of EMT after CCN3 treatment was demonstrated by a shift from the expression of epithelial markers to mesenchymal markers in DU145 cells (Figure 4C–4E). Moreover, CCN3 treatment significantly increased the numbers of DU145 cells migrating across the wound (Figure 4F and 4G). The cell invasion assay also revealed pro-invasive CCN3 activity (Figure 4H and 4I). These results suggest that CCN3 directly promotes EMT in PCa cells.

### CCN3 induces FAK/Akt/HIF-1α activation, stimulates twist expression and promotes EMT in PCa cells

**Figure 5:**
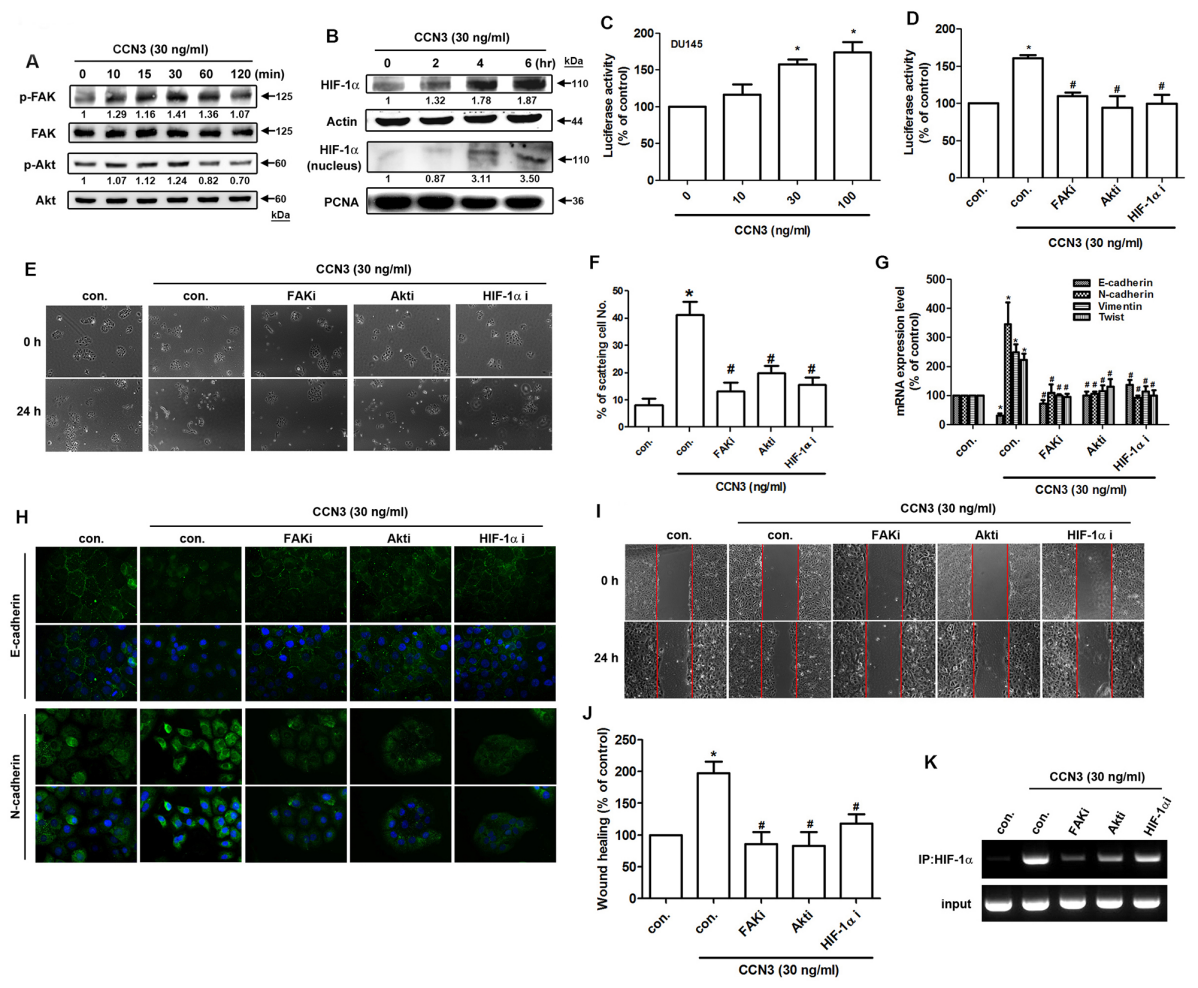
CCN3 triggers FAK/Akt/HIF-1α pathway to induce Twist expression and promotes EMT in PCa cells **(A)** DU145 cells were incubated with CCN3 (30 ng/mL) for the indicated times, and phosphorylation of FAK and Akt was determined by western blot analysis. **(B)** DU145 cells were incubated with CCN3 (30 ng/mL) for the indicated times. The total protein and nuclear extract were prepared, and HIF-1α expression levels were determined by western blot analysis. Actin and PCNA were used as internal control. **(C)** DU145 cells were transfected with an HRE promoter reporter plasmid for 24 h, then incubated with CCN3 for 24 h at indicated doses and luciferase activity was measured. **(D)** DU145 cells were transfected with an HRE promoter reporter plasmid for 24 h, followed by treatment with FAKi (10 μM), Akti (1 μM) or HIF1-α i (1 μM) for 30 min. The cells were then incubated with CCN3 (30 ng/mL) for 24 h, and luciferase activity was measured. **(E)** DU145 cells were treated with FAKi (10 μM), Akti (1 μM) or HIF1-α i (1 μM) for 30 min and then incubated with CCN3 (30 ng/mL) for 24 h. The cells were photographed to analyze scattering and morphology. **(F)** The quantitative analysis of scatter assay which was performed in Figure 3A. **(G)** DU145 cells were treated as described (Figure 4E), total RNA was extracted, and expression levels of E-cadherin, N-cadherin, vimentin, and Twist were examined by qPCR. **(H)** DU145 cells were treated as (Figure 4E) described, the cells were stained with E-cadherin and N-cadherin antibodies and analyzed by fluorescence microscopy. Nuclei were counterstained with DAPI. Representative microscopy images are shown. **(I and J)** DU145 cells were treated as (Figure 4E) described, and *in vitro* migration was measured by wound healing assay. The migrating cells were photographed and counted. **(K)** DU145 cells were pretreated with FAKi (10 μM), Akti (1 μM) or HIF1-α i (1 μM) for 30 min and then incubated with CCN3 (30 ng/mL) for 24 h. Chromatin immunoprecipitation was performed with HIF1-α antibody. One percent of immunoprecipitated chromatin was assayed to verify equal loading (input). Results are expressed as the mean ± S.E.M. *p < 0.05 compared with control, ^#^p < 0.05 compared with CCN3-treated group.

A previous study indicated that CCN3 stimulates intracellular signaling pathways through cell surface integrin receptors [23]. The integrin outside-in signaling triggers variant pathways such as FAK and PI3K/Akt [24]. Our data showed that treatment of PCa cells with CCN3 (30 ng/mL) increased the phosphorylation of FAK and Akt signaling proteins (Figure 5A). Moreover, CCN3 treatment increased HIF-1α expression and nuclear accumulation in a time-dependent manner (Figure 5B). Using the hypoxia responsive element (HRE)-luciferase activity assay, we confirmed an increase in HIF-1α activity (Figure 5C). Furthermore, pretreatment with inhibitors of FAK, Akt, and HIF-1α significantly inhibited CCN3-induced HIF-1α activity (Figure 5D). These pathway inhibitors were applied to CCN3-induced EMT in PCa cells. Pretreatment with indicated inhibitors reversed CCN3-induced cell scattering, changes in EMT markers, and migratory potential of PCa cells (Figure 5E-5J). A previous study has shown that HIF-1α regulates the expression of Twist through the HRE located in the Twist proximal promoter, and induces EMT and metastatic phenotypes *in vitro* and *in vivo* [25]. In our study, chromatin immunoprecipitation results confirmed that CCN3 mediated the binding of HIF-1α to the HRE element located in the distal promoter region of Twist, and this phenomenon was reversed by treatment with FAK, Akt, and HIF-1α inhibitors (Figure 5K). These results demonstrate that CCN3 promotes EMT through the FAK/Akt/HIF-1α signaling pathway and induces Twist expression in PCa cells.

### CCN3 is positively correlated with Twist expression in PCa specimens

**Figure 6:**
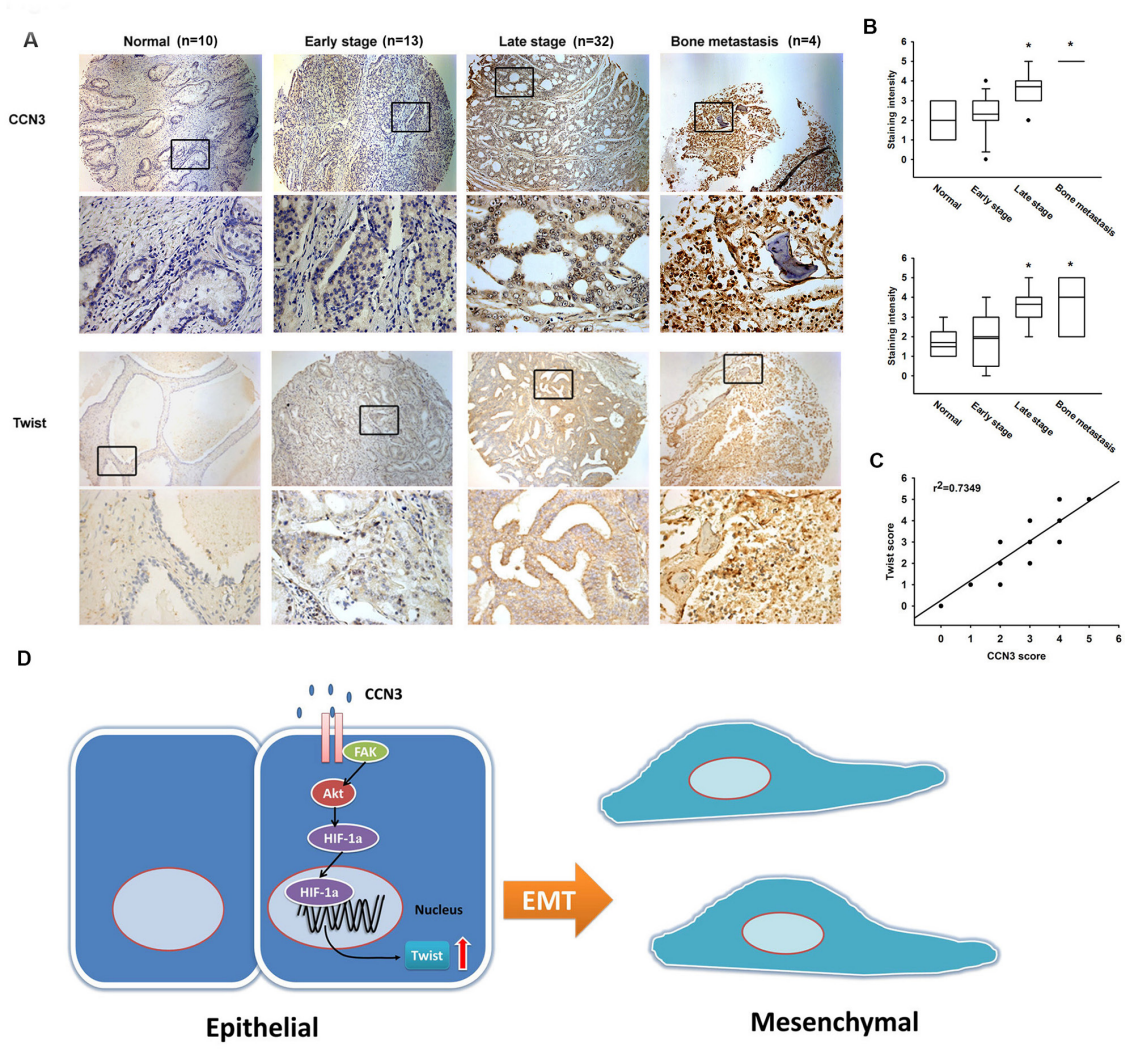
Prostate cancer specimens show significant correlations between CCN3, Twist expression and tumor progression **(A)** Tumor specimens were stained with CCN3 and Twist antibodies by IHC staining. The stained specimens were photographed by optical microscope. **(B)** The IHC stain intensities of CCN3 and Twist were scored from 1-5 to quantify the expression levels of CCN3 and Twist in different stages. **(C)** IHC stain scores of CCN3 and Twist were paired from the same specimens and the correlation between CCN3 and Twist expression levels was shown by linear regression in prostate cancer specimens. **(D)** Diagrammatic model for the molecular mechanism of CCN3-promoting EMT in prostate cancer.

Our data indicate that CCN3 induces Twist expression in PCa cells. Therefore, it is important to investigate the correlation between CCN3 and Twist in clinical specimens, and their prognostic relevance. Higher expression levels of both CCN3 and Twist were found to be associated with higher clinical pathologic stages, with strong staining in clinical samples of PCa bone metastases (Figure 6A and 6B). Furthermore, CCN3 expression was positively correlated with Twist expression in PCa specimens (Figure 6C), suggesting that CCN3 is linked with Twist expression and tumor metastasis in PCa.

## DISCUSSION

A substantial body of evidence indicates that cancer cells undergo EMT to acquire the ability to invade the surrounding ECM. Thus, EMT is a critical step in tumor metastasis. Recent reviews reveal that EMT pathways could serve as a promising therapeutic target [26, 27]. In the present study, we provide evidence showing that CCN3 induces EMT and thus promotes metastasis in PCa cells.

A previous study has shown CCN3 overexpression in metastatic melanoma cells compared with primary tumor cells, and overexpression of CCN3 improves adhesion to ECM proteins by regulating integrin expression [28]. This process induces the change in cancer cells to an aggressive phenotype. In addition, CCN3 regulates cancer cell mobility, which correlates with tumor metastasis. For instance, in renal cell carcinoma, CCN3 promotes cell migration and invasion by upregulating ICAM-1 and COX-2 expression [29, 30]. The data from our orthotopic model show that the knockdown of CCN3 expression dramatically abolishes tumor growth and metastasis, especially bone metastasis. Many studies show that CCN3 and other CCN family members play a crucial role in bone homeostasis [31]. Dysfunction or dysregulation of CCN3 causes tumorigenesis and contributes to bone metastasis. Previous studies have discussed the crucial role of CCN3 in osteolytic bone metastasis [20, 32]. Our orthotopic tumor model provides strong support for the contention that CCN3 is responsible for bone-tropic PCa metastasis. Here, we found that CCN3 promoted Twist expression in prostate cancer cells. In a previous study, Twist promoted bone metastasis by regulating prostate cancer cell-mediated bone remodeling [33]. The other EMT transcription factor ZEB1 has also been implicated in the promotion of osteolytic bone metastases in breast cancer [34]. These findings suggest that CCN3 may regulate bone metastasis through bidirectional mechanisms.

The prognostic value of CCN3 differs according to type of tumor. High CCN3 expression correlates with a poor prognosis in renal and prostate carcinomas, Ewing tumors, osteosarcomas and melanoma, whereas in gliomas, chronic myeloid leukemia, malignant adrenocortical tumors and neuroblastoma, high CCN3 expression is associated with a good prognosis [31, 35]. This evidence demonstrates that the biological properties of CCN3 depend upon the cellular context. The CCN family of cysteine-rich matricellular proteins contains six members, which play a crucial role in regulating various cell functions such as migration and adhesion during pathophysiological processes [36]. CCN1 induces EMT and stem cell-like traits in pancreatic cancer [37]. Moreover, CCN1 also plays a central role in metastatic osteosarcoma by upregulating Twist expression [38]. Inhibition of CCN6 expression promotes cell invasion and EMT in breast epithelial cells by upregulating mesenchymal markers (snail and ZEB1) and by suppressing the epithelial marker, E-cadherin [39]. It has previously been reported that CCN3 overexpression regulates actin reorganization by increasing the activity of the small GTPase Rac1, which promotes PCa cell migration [40]. Interestingly, a constitutively active Rac1 mutant induces EMT in transformed keratinocytes [41]. These studies reveal how CCN3 promotes EMT. A previous review indicates that CCN3 is a key player in stem cell regulation [18]. The common features of EMT and stem cell plasticity implicate the crucial role played by CCN3 in tumor progression. CCN3 has been shown to be a positive regulator of EMT in pancreatic cancer [42]. However, the mechanism of this regulation remains unclear. We therefore explored the mechanism of CCN3 action in PCa.

Hypoxia accompanies tumor growth and is a common feature in many types of solid tumors. Study evidence reveals close correlations between CCN family members and environmental hypoxia. For instance, CCN1, CCN2 and CCN3 are upregulated in hypoxic conditions through HIF-1α activation [43, 44]. These family members have been identified as angiogenic factors [45–47], so it is not surprising that they are highly regulated by hypoxia. The most important transcription factor involved in hypoxic responses is HIF-1α, with several studies demonstrating its pivotal role in EMT regulation [48]. Our results show that CCN3 induces EMT through HIF-1α activation , which suggests that HIF-1α may be a central component in EMT regulation in PCa. We also found that CCN3 promotes Twist expression by activating HIF-1α. A previous study has shown that HIF-1α regulates Twist gene expression by binding directly to the HRE located in the proximal promoter region of Twist [25]. However, our results indicate that HIF-1α binds to the HRE located in the distal promoter region of Twist. This difference may be due to the different types of cancer cells. The analysis of clinical specimens also shows a correlation between the expression levels of CCN3 and Twist, which agrees with the *in vitro* results. This finding underscores the potential of CCN3 as a promising prognostic marker in PCa metastasis.

Bone metastasis remains inevitable in prostate cancer progression. The drugs used in clinical therapy only attenuate the growth of bone metastases and bone pain. This work provides novel insights into CCN3 that may encourage its development as a therapeutic target, as a means of preventing bone metastasis in prostate cancer.

## MATERIALS AND METHODS

### Materials

Protein A/G beads, anti-mouse and anti-rabbit IgG-conjugated horseradish peroxidase, mouse monoclonal or rabbit polyclonal antibodies specific for CCN3 (SC-136967), Twist (SC-81417), N-cadherin (ab76057), E-cadherin (ab40772), p-FAK (SC-11765-R), FAK (SC-932), p-Akt (SC-16646-R), Akt (SC-5298), HIF-1α (ab1), and β-actin (SC-130656) were purchased from Santa Cruz Biotechnology (Santa Cruz, CA, USA) or Abcam (Cambridge, UK). Recombinant human CCN3 was purchased from PeproTech (Rocky Hill, NJ, USA). All other chemicals were obtained from Sigma–Aldrich (St Louis, MO).

### Cell culture

All cell lines were obtained from the Bioresource Collection and Research Center (BCRC, Hsinchu, Taiwan). PC3 and DU145 are androgen-independent cell lines derived from bone and brain metastasis, respectively. These cell lines were cultured in F-12 or α-MEM medium, respectively, supplemented with 20 mM HEPES, 10% FBS, 2 mM glutamine, 100 U/mL penicillin, and 100 μg/mL streptomycin (Invitrogen, Carlsbad, CA). The PZ-HPV-7 cell line is a virus-transformed normal human prostate epithelial cell and cultured in keratinocyte serum-free medium (Invitrogen, Carlsbad, CA). All cell lines were maintained at 37°C in a 5% CO_2_ atmosphere.

### Preparation of conditioned media

PC3 cells (2×10^6^) were grown overnight in 100-mm culture dishes in cell culture medium. After 2 washes with phosphate-buffered solution (PBS), cells were incubated in 1% FBS in F-12 medium for 48 h before collection of the CM. To normalize for differences in cell density due to proliferation during the culture period, cells from each plate were collected and the total DNA content/plate was determined (spectrophotometric absorbance, 260 nm). The CM was then normalized for DNA content between samples by adding F-12 medium.

### Wound healing assay

Cells (1 × 10^5^ cells/well) were seeded in 12-well plates in culture medium. After incubating the cells for 24 h, the confluent monolayer of culture was scratched with a pipette tip, and wound healing was visualized by microscopy. The migrating cells were photographed and counted. The rate of wound closure was observed at the indicated times as described in the Figure legends.

### Cell invasion assay

All cell invasion assays were performed using Transwell inserts (8-μm pore size; Costar, NY) in 24-well dishes. The wells were pre-coated with 20 μl Matrigel (25 mg/50 mL; BD Biosciences, Bedford, MA) to form a continuous, thin layer. Stable cells (1 × 10^4^ in 200 μl of medium containing 1% FBS) were then seeded in the upper chamber of the Transwell and 300 μl of the same medium were placed in the lower chamber. To assess the EMT-promoting effect of CCN3, the same medium containing varying concentrations of CCN3 (PeproTech, Rocky Hill, NJ, USA) was placed in the lower chamber. Each experiment was performed with triplicate wells and repeated at least 3 times.

### Immunofluorescence microscopy

Cells grown on glass coverslips were rinsed with PBS and fixed in 3.7% formaldehyde for 10 min at room temperature. Cells were washed 3 times with PBS and blocked with 4% BSA for 15 min. Cells were then incubated with the indicated primary antibody (1:100) for 1 h at room temperature, washed again, and incubated with FITC-conjugated secondary antibody for 1 h. Finally, cells were washed, mounted, and photographed with a Leica DMI 3000 System.

### Scatter assay

To monitor EMT changes *in vitro*, the cell scatter assay was performed on DU145 cells, according to previously described methods [21]. Cells were seeded in a 24-well plate at a density of 0.5 × 10^4^ cells/well. The cells formed small colonies after 48 h. Subsequently, the cells were treated as indicated under different conditions as described in the Figure legends and incubated for a further 24 h. Finally, cells were fixed in 4% PFA and photographed by microscope-mounted camera. The quantitative analysis of scatter assay was calculated by percentage of scattering cell numbers/ total cell numbers.

### Reporter assay

The DU145 cells were transfected with HRE reporter plasmid using Lipofectamine 2000 (Invitrogen), according to the manufacturer’s instructions. After 24 h, the cells were treated with inhibitors for 30 min. This was followed by the addition of CCN3 (30 ng/mL) or vehicle, and the cells were incubated further for 24 h. Cell extracts were then prepared, and luciferase and β-galactosidase activities were measured.

### CCN3 knockdown and overexpression in PCa cell lines

The lentiviral expression system for CCN3 knockdown and overexpression was purchased from the National RNAi Core Facility (RNAi Core, Academia Sinica, Taiwan). The CCN3 shRNA plasmid (clone ID: TRCN0000107130) was selected to knock down gene expression. The CCN3 overexpression plasmid was constructed by introducing full-length CCN3 ORF into pLKO_AS2.puro (RNAi Core). An empty vector was used as a negative control. The highly aggressive PCa cell line PC3 was transfected with the CCN3 shRNA plasmid, and the less aggressive PCa cell line DU145 was transfected with the CCN3 overexpression plasmid. The cells were puromycin-selected and the surviving cells were used as stable gene-modified cell lines. DU145 and PC3 cell lines that stably expressed luciferase were established before transfection with the CCN3 overexpression vector, CCN3 shRNA vector, or the control vector and the *in vivo* orthotopic model was analyzed using the *In Vivo* Imaging Systems (IVIS, Xenogen, UK).

### Western blot analysis

The cellular lysates were prepared, the proteins were resolved by SDS–PAGE, and then transferred to Immobilon polyvinyldifluoride (PVDF) membranes. The blots were blocked with 4% BSA for 1 h at room temperature and then probed with rabbit anti-human antibodies against FAK, p-FAK, Akt, p-Akt, HIF-1α, E-cadherin, N-cadherin, or Twist (1:1000) for 1 h at room temperature. After three washes, the blots were subsequently incubated with a donkey anti-rabbit peroxidase-conjugated secondary antibody (1:1000) for 1 h at room temperature. The protein bands were visualized by enhanced chemiluminescence using ImageQuant LAS 4000 (GE Healthcare Life Sciences, Little Chalfont, UK).

### Quantitative real-time PCR

Quantitative real-time polymerase chain reaction (qPCR) analysis was performed using the Taqman one-step PCR Master Mix (Applied Biosystems, Foster City CA); 100 ng of total cDNA was added per 25-μl reaction with sequence-specific primers and Taqman probes. All target gene primers and probes were purchased commercially (Applied Biosystems, CA). β-actin was used as an internal control. Quantitative RT-PCR assays were performed in triplicate on a StepOnePlus sequence detection system. The PCR conditions were: 10 min polymerase activation at 95°C (initial denaturation), followed by 40 cycles of 95°C for 15 sec and 60°C for 60 sec. The threshold for detection was set above the non-template control background and within the linear phase of target gene amplification to calculate the cycle number at which the transcript is detected (denoted as C_T_).

### Chromatin immunoprecipitation assay

Chromatin immunoprecipitation analysis was performed as described previously [49]. The DNA immunoprecipitated by anti-HIF-1α antibody was purified and extracted with phenol–chloroform, then subjected to PCR analysis. The primers designed for human Twist promoter region were selected as previously described [25]. Forward primer with the sequence 5′-TACTCCAGCGCGGTGCACAAAACT-3′ and reverse primer with the sequence 5′- AACGAAGAGCCCCAAAGAGGGTGT-3′ were used to amplify the human Twist promoter region (-1383 to -1215). PCR products were then resolved by running on 1.5% agarose gel and visualized under ultraviolet light.

### Histology and immunohistochemistry (IHC)

For investigating CCN3 expression in clinical specimens, human prostate cancer tissue array (PR956 and T195a) was purchased from Biomax (Rockville, MD). All specimens in these arrays contain 49 cases of PCa tissue, plus 10 normal prostate tissue cases. The PCa tissues contained 2 stage I, 11 stage II, 27 stage III, 5 stage IV and 4 bone metastasis tissue specimens, as defined by the histological grading system provided by Biomax. For *in vivo* IHC staining in the orthotopic model, tumor, lung, liver, and leg samples collected from sacrificed mice were fixed in 4% paraformaldehyde in PBS for at least 72 h, and dehydrated in increasing concentrations of ethanol. The legs were decalcified in 10% EDTA for 14 days. All samples were then embedded in paraffin. Sections (5-μm thick) of paraffin-embedded tissue were placed on glass slides, rehydrated, incubated with 3% hydrogen peroxide to quench endogenous peroxidase activity, and then blocked by 3% BSA incubation in PBS. Sections were incubated with the primary mouse polyclonal anti-human CCN3 and Twist antibody at 1:100 dilutions, at 4°C overnight. After three PBS washes, samples were incubated with a 1:100 dilution of biotin-labeled goat anti-mouse IgG secondary antibody. The bound antibodies were detected by ABC Kit (Vector Laboratories, Burlingame, CA). Slides were stained with the chromogen diaminobenzidine, washed, counterstained with Delafield’s hematoxylin, dehydrated, treated with xylene, and finally mounted. Stained specimens were photographed by microscope. The stain intensity was scored from 0-5 to quantify the expression levels observed in the photographs, using the following scoring approach: 0 = no staining or unspecific staining of tumor cells; 1 = very weak (intensity) of tumor cells; 2 = weak staining of tumor cells; 3 = moderate staining of tumor cells; 4 = strong staining of tumor cells; 5 = very strong staining of tumor cells. A pathologist evaluated the spectrum of staining intensity in all the samples and then arbitrarily categorized the staining intensity into the various scores based on the overall staining.

### Orthotopic animal model and imaging

This study were approved by the China Medical University Institutional Animal Care and Use Committee (reference number 103-222-NH). All experimental procedures were approved by the Institutional Animal Care and Use Committee. The ventral prostate of male nu/nu mice (6–8 weeks old) was exposed by midventral incision and injected with 5× 10^5^ DU145 Neg and DU145 shCCN3 cells suspended in 50 μL PBS. One week after injection, surgical staples were removed, and the tumor growth and local metastasis were monitored using IVIS Imaging System. The mice were sacrificed 35 days later. Lungs, livers, and four limbs of the sacrificed mice were dissected and monitored using IVIS Imaging System. The percentage of lung metastasis was conducted by (No. of positive signal lungs / No. of total lungs) using IVIS Imaging System. The other metastasis were calculated as well as lungs.

### Statistical analysis

All values are expressed as the mean ± S.E.M. The significance of any differences between the experimental groups and controls were assessed by Student’s *t* test. The difference was considered significant if the *p* value was < 0.05.

## Abbreviations

EMT: epithelial-mesenchymal transition
PCa: prostate cancer
NOV: Nephroblastoma overexpressed, also known as CCN3
PVDF: polyvinyldifluoride
HIF-1α: hypoxia inducible factor-1α
IHC: immunohistochemistry;
qPCR: quantitative real-time PCR
